# Supplementary material for: Tumor Androgen Receptor Protein Level Is Positively Associated with a Better Overall Survival in Melanoma Patients
Source: Genes (Basel). 2023 Jan 28;14(2):345. doi: 10.3390/genes14020345 (PMC9957358; doi:10.3390/genes14020345)
Supplement: Supplementary file 1 [file genes-14-00345-s001.zip › genes-2122360-supplementary.pptx]

## Slide 1
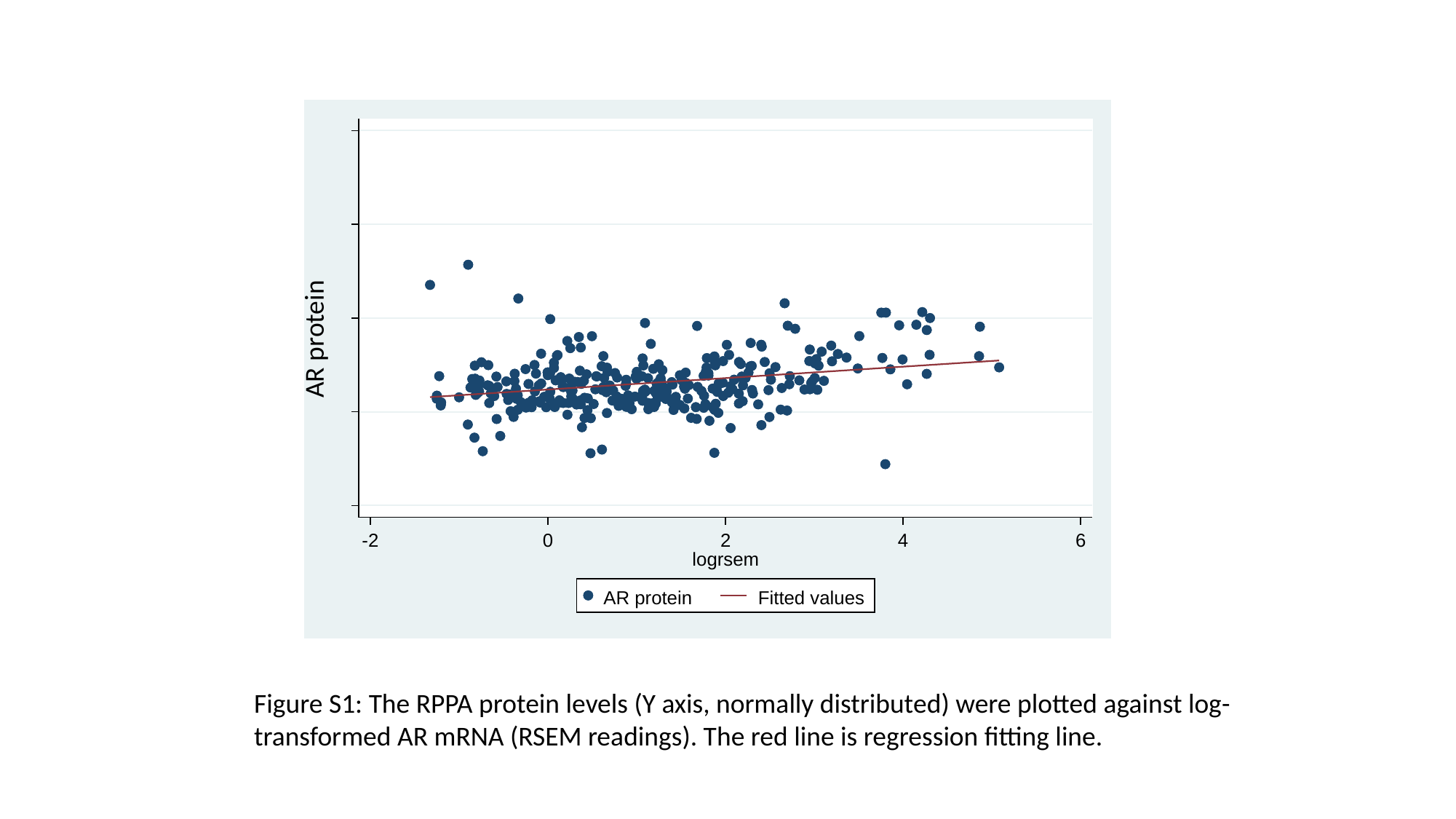

AR protein
Figure S1: The RPPA protein levels (Y axis, normally distributed) were plotted against log-transformed AR mRNA (RSEM readings). The red line is regression fitting line.

## Slide 2
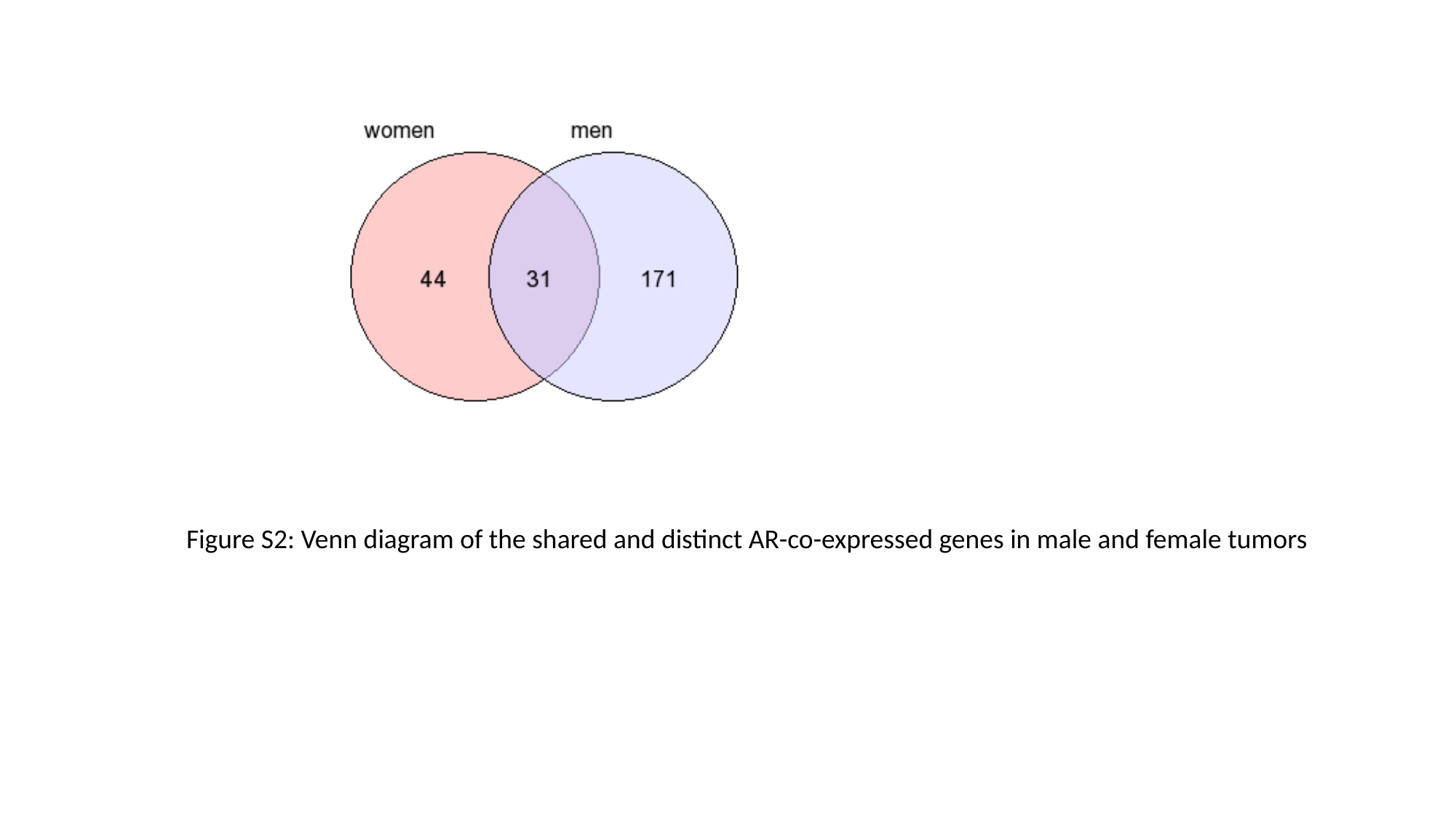

Figure S2: Venn diagram of the shared and distinct AR-co-expressed genes in male and female tumors
